# Explainable machine learning model for classifying atherosclerotic cardiovascular disease in patients with metabolic dysfunction-associated steatotic liver disease

**DOI:** 10.3389/fendo.2025.1684558

**Published:** 2025-10-30

**Authors:** Zhengliang Li, Xiaokai Chen, Linlin Ren, Banghui Wang, Shimiao Ruan, Juan Wang, Wenzhong Zhang

**Affiliations:** ^1^ Department of Cardiology, The Affiliated Hospital of Qingdao University, Qingdao, Shandong, China; ^2^ Department of Gastroenterology, The Affiliated Hospital of Qingdao University, Qingdao, Shandong, China; ^3^ Department of Emergency, The Affiliated Hospital of Qingdao University, Qingdao, Shandong, China

**Keywords:** metabolic dysfunction-associated steatotic liver disease, atherosclerotic cardiovascular disease, composite metabolic index, machine learning, SHAP interpretability model

## Abstract

**Background:**

Cardiovascular disease (CVD) is the leading cause of mortality in patients with metabolic dysfunction-associated steatotic liver disease (MASLD), yet traditional risk predictors remain limited in clinical practice.

**Objective:**

To develop machine learning (ML) models for classifying prevalent atherosclerotic cardiovascular disease (ASCVD) risk in MASLD patients, and to enhance model interpretability using SHapley Additive exPlanations (SHAP). Methods: This retrospective study included 590 MASLD patients diagnosed at the Affiliated Hospital of Qingdao University between December 2019 and December 2024. Patients were randomly divided into a training set (n=413) and a validation set (n=177), and further stratified based on ASCVD status. Least absolute shrinkage and selection operator (LASSO) regression was used for feature selection. Six ML models were developed and evaluated using sensitivity, specificity, accuracy, area under the receiver operating characteristic curve (AUC), and F1 score. SHAP analysis was performed to interpret feature contributions.

**Results:**

ASCVD was present in 434 of 590 patients (73.6%). The Gradient Boosting (GB) model achieved the best performance, with AUCs of 0.918 (95% CI: 0.890–0.944) in the training set and 0.817 (95% CI: 0.739–0.883) in the validation set. SHAP analysis identified the top predictors as the Cholesterol–HDL–Glucose (CHG) index, Castelli Risk Index II (CRI-II), lipoprotein(a) [Lp(a)], serum creatinine (Scr), and uric acid (UA).

**Conclusion:**

The GB model demonstrated strong high accuracy in identifying existing ASCVD in MASLD patients and may serve as a useful tool for early risk stratification in clinical settings.

## 1 Introduction

Non-alcoholic fatty liver disease (NAFLD) is a chronic metabolic stress–related liver disease that arises in genetically predisposed individuals due to overnutrition and insulin resistance (IR) (1). With the global rise in obesity and type 2 diabetes, the diagnostic criteria for this condition have undergone significant revisions. In 2020, an international expert panel proposed renaming the disease as metabolic dysfunction-associated fatty liver disease (MAFLD) (2), reflecting its underlying pathophysiology more accurately. In 2023, the European Association for the Study of the Liver (EASL) further updated the terminology to metabolic dysfunction-associated steatotic liver disease (MASLD), emphasizing the central role of metabolic and cardiovascular risk factors in its diagnosis (1). Recent epidemiological studies indicate that MASLD has become one of the most prevalent chronic liver diseases in China, with a continuously rising incidence (3).

Atherosclerotic cardiovascular disease (ASCVD) is one of the leading causes of death and disability worldwide (4–7). Its pathogenesis is closely linked to atherosclerosis and metabolic dysfunction. MASLD and ASCVD share multiple metabolic risk factors, and accumulating evidence suggests that the both presence and severity of MASLD are strongly associated with increased ASCVD risk (8, 9), Moreover, ASCVD is a major cause of mortality in patients with MASLD (10). Therefore, developing reliable and effective classification of prevalent ASCVD tools is critical for the early identification and intervention in individuals at high risk MASLD populations.

Currently, cardiovascular disease (CVD) risk assessment primarily relies on traditional indicators such as age, sex, smoking status, blood pressure, and high-density lipoprotein cholesterol (HDL-C) levels (11). Although widely used in clinical practice, these models have notable limitations. While HDL-C is a well-established inverse predictor of ASCVD events (12), its discriminative ability in identifying ASCVD among MASLD patients is limited. Such models often fail to account for the combined effects of dyslipidemia and impaired glucose regulation. In recent years, composite metabolic indices such as the cholesterol–HDL–glucose (CHG) index and Castelli’s Risk Index II (CRI-II) have been proposed to better capture the impact of metabolic disturbances on cardiovascular risk (13).

Machine learning (ML), as an emerging modeling approach, offers strong capabilities in handling complex interactions and nonlinear relationships, and has been widely applied in the development of medical prediction models (14–17). For instance, Durán et al. (18) demonstrated nonlinear associations between gastric microbiota and proton pump inhibitor exposure, while Kha et al. (19) employed the Extreme Gradient Boosting (XGBoost) model to identify​ interactions ​among oral diabetes medications in patients with diabetes. Despite these powerful capabilities and increasing applications, a notable limitation of ML models remains their limited interpretability, ​often leading them to be characterized as “black box” models (20). SHapley Additive exPlanations (SHAP), a widely adopted interpretability framework in recent years, improves model transparency and clinical acceptability by quantifying the contribution of each feature to the prediction output (21, 22).

Therefore, this study aimed to develop multiple ML models to identify existing ASCVD in patients with MASLD, and to incorporate the SHAP method for model interpretation, thereby providing an accurate, efficient, and interpretable tool to support clinical decision-making.

## 2 Methods

**Figure 1:**
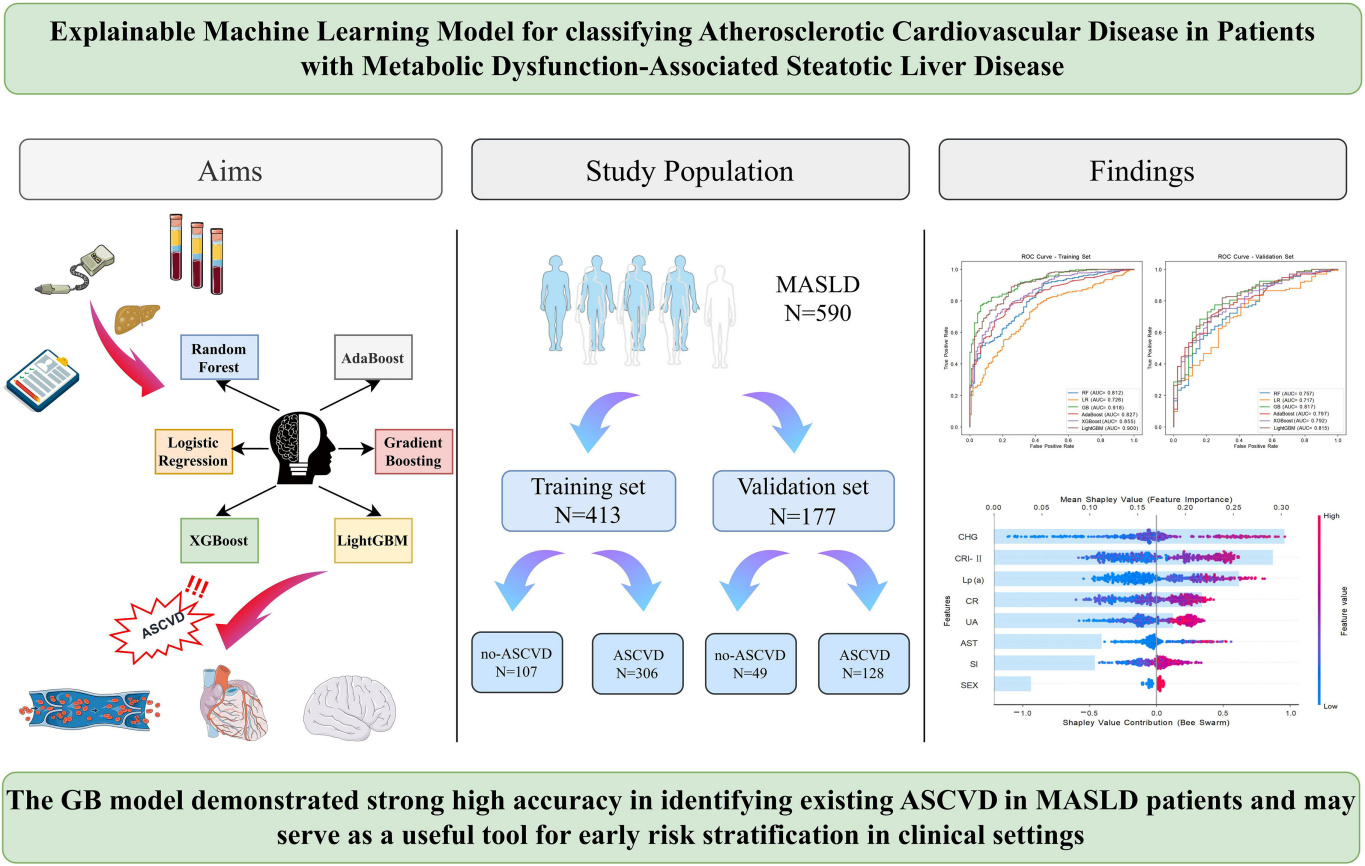
Workflow diagram of this study.

This study collected blood samples, basic medical history information, and ultrasound reports from patients, and conducted ML based on population characteristics to evaluate model performance and establish a model capable of effectively identifying MASLD patients at risk of ASCVD. The entire research workflow is summarized in **Figure 1**.

### 2.1 Study population

A total of 590 inpatients diagnosed with MASLD were retrospectively enrolled from the Affiliated Hospital of Qingdao University between December 2019 and December 2024. Written informed consent was obtained from all participants. The study protocol was approved by the Ethics Committee of the Affiliated Hospital of Qingdao University.

### 2.2 Inclusion and exclusion criteria

Inclusion criteria were as follows (1): Hepatic steatosis confirmed by standard abdominal ultrasonography (23). (2) Diagnosis of MASLD, defined as hepatic steatosis accompanied by at least one metabolic cardiovascular risk factor, including obesity, hypertension, prediabetes or a history of type 2 diabetes mellitus, hypertriglyceridemia, or low HDL-C levels.

Exclusion criteria included: (1) History of excessive alcohol consumption (≥140 g/week for men or ≥70 g/week for women); (2) History of viral hepatitis, liver cirrhosis, autoimmune liver disease, or drug-induced liver injury; (3) Use of antiplatelet or lipid-lowering medications; (4) History of coronary intervention or coronary artery bypass grafting (CABG); (5) Presence of other cardiac diseases;

(6) Severe renal insufficiency, malignancy, autoimmune disorders, acute or chronic infectious diseases, or major cerebrovascular disease.

The diagnosis of ASCVD was based on the 2013 ACC/AHA Guideline on the Treatment of Blood Cholesterol to Reduce Atherosclerotic Cardiovascular Risk in Adults (10), and included any of the following conditions: congestive heart failure, stable or unstable angina, acute myocardial infarction, ischemic stroke, or peripheral atherosclerosis.

### 2.3 Grouping method

All MASLD patients were classified into two groups based on the presence or absence of ASCVD: the MASLD-only group and the MASLD+ASCVD group. Subsequently, the entire cohort was randomly divided into a training set (n = 413) and a validation set (n = 177) in a 7:3 ratio for model development and validation.

### 2.4 Data collection and feature construction

Basic patient information was obtained from the electronic medical record system, including sex, age, height, weight, smoking and alcohol consumption history. Laboratory parameters included fasting blood glucose (FBG), alanine aminotransferase (ALT), aspartate aminotransferase (AST), triglycerides (TG), total cholesterol (TC), lipoprotein(a) [Lp(a)], HDL-C, low-density lipoprotein cholesterol (LDL-C), serum creatinine (Scr), cystatin C (CYSC), uric acid (UA), and blood cell count–related indices.

Based on the above data, several composite metabolic indicators were calculated to better reflect patients’ metabolic status, including body mass index (BMI), sarcopenia index (SI), CHG index, Castelli’s Risk Index I and II (CRI-I, CRI-II), atherosclerosis index (AIP), triglyceride–glucose (TyG) index, and the BMI-adjusted TyG index (TyG-BMI).

Data preprocessing and feature engineering were conducted in a Python 3.8 environment. The Pandas library (v1.3.3) was used for data loading, cleaning, and structural formatting. Numerical computation and missing value imputation were performed using NumPy (v1.21.2). Complete technical specifications are documented in Appendix A. To reduce feature dimensionality, enhance modeling efficiency, and control multicollinearity, least absolute shrinkage and selection operator (LASSO) regression with L1 regularization was applied for feature selection. The final feature set was determined exclusively by the LASSO regression without clinical judgment intervention.​​ Ten-fold cross-validation was performed using the LassoCV module in the Scikit-learn library to automatically determine the optimal regularization parameter λ. Variables with non-zero coefficients were retained as input features for model construction. A regularization path plot illustrating coefficient shrinkage during LASSO selection was generated using Matplotlib (v3.4.3).

### 2.5 Machine learning model construction and hyperparameter optimization

After feature extraction and variable construction, six commonly used ML algorithms were developed based on the training set: Random Forest (RF), Logistic Regression (LR), Gradient Boosting (GB), Adaptive Boosting (AdaBoost), XGBoost, and Light Gradient Boosting Machine (LightGBM) (24). The selection of the six machine learning approaches was guided by the following considerations: (a) Small-sample robustness:​ Tree-based ensembles (RF, GB, AdaBoost, XGBoost, LightGBM) mitigate overfitting through regularization and ensemble mechanisms, while LR provides stable baselines for low-dimensional patterns; (b) Interpretability:​ LR enables direct coefficient interpretation, and tree-based models offer native feature importance outputs. (c) Computational efficiency:​ All ML demonstrate fast convergence on moderate-sized datasets, avoiding complex models requiring large-scale data (25, 26). Model development and training were conducted in a Python environment using mainstream open-source libraries, including Scikit-learn, XGBoost (v1.5.1), and LightGBM (v3.3.2).

To enhance model generalizability, all models were trained using a Pipeline framework combined with 10-fold cross-validation. Hyperparameter tuning was performed using the RandomizedSearchCV method. Model inputs were the key features selected via LASSO regression, and the output was a binary classification indicating the presence or absence of ASCVD.

To evaluate model performance, the following metrics were calculated on the validation set: area under the receiver operating characteristic curve (AUC), sensitivity, specificity, accuracy, positive predictive value (PPV), and F1 score. AUC was used as the primary indicator of discriminatory ability, while the F1 score was particularly emphasized to assess precision–recall trade-offs in the presence of class imbalance.

In addition, normalized confusion matrices were generated to visualize the classification ability of each model for positive and negative cases. Based on the overall performance across metrics, the best-performing model was selected for subsequent interpretability analysis.

Meanwhile, we selected the Prediction for ASCVD Risk in China (China-PAR project) model as the primary benchmark because it represents the current standard for ASCVD risk assessment in Chinese populations (27). It is important to note that while China-PAR was developed in general population cohorts, our study specifically targets the MASLD subpopulation.

### 2.6 Model interpretability analysis (SHAP)

To improve the transparency and interpretability of the machine learning model, SHAP was applied to the best-performing model for both global and individual-level interpretation. SHAP is based on the Shapley value concept from game theory and assigns each feature a contribution value for every individual prediction, thereby enabling transparent explanations of complex, nonlinear models.

At the global level, a SHAP feature importance plot was generated to rank the input variables according to their average absolute SHAP values, which reflect the mean magnitude of impact each feature has on the model’s output. Higher values indicate greater overall influence on predictions.

A SHAP summary plot was then constructed to visualize how individual feature values contributed to model outputs across the entire dataset. Each point on the plot represents the SHAP value of a specific feature for one patient. The color gradient from red to blue indicates high to low feature values, while the distribution along the x-axis shows the direction and magnitude of each feature’s effect on the prediction.

To further investigate nonlinear effects and feature interactions, SHAP dependence plots were generated. These plots visualize the marginal effects of predictors across their value ranges, revealing threshold effects and nonlinear relationships with prevalent ASCVD status.

At the individual level, SHAP force plots were created for two representative patients randomly selected from the cohort. These visualizations explain how each feature contributes to a specific prediction. In the force plots, red bars represent features that push the prediction toward the positive class (MASLD+ASCVD), while blue bars represent features that push it toward the negative class. The sum of these contributions, starting from the model’s base value, yields the final predicted probability, illustrating a clear, feature-wise explanation path.

All SHAP analyses were performed in Python using the SHAP library (v0.40.0), with visualizations generated using Matplotlib and Plotly. The incorporation of SHAP not only enhanced model transparency but also provided valuable insights into high-risk features, thereby improving both clinical utility and model credibility.

### 2.7 Statistical analysis

All statistical analyses and visualizations were performed using R software (version 4.3.3) and SPSS software (version 27.0.0). Continuous variables were described as mean ± standard deviation (SD) for normally distributed data, or as median with interquartile range (IQR) for non-normally distributed data. Categorical variables were expressed as counts (n) and percentages (%).

Comparisons between groups for categorical variables were conducted using the chi-square (χ²) test. For continuous variables, independent samples t-tests were applied for normally distributed data, while nonparametric tests (e.g., Mann–Whitney U test) were used for skewed distributions. All statistical tests were two-sided, and a p-value < 0.05 was considered statistically significant.

## 3 Results

### 3.1 Comparison of baseline characteristics

A total of 590 patients with MASLD were enrolled in the study, including 413 cases assigned to the training set and 177 to the validation set. Within the training set, patients were further stratified based on ASCVD status into the MASLD-only group (n = 107) and the MASLD+ASCVD group (n = 306).

Significant differences were observed between the two groups in several clinical and laboratory parameters. The MASLD+ASCVD group showed higher proportions of male patients and elevated levels of FBG, ALT, TG, TC, Lp(a), LDL-C, Scr, UA, CHG index, CRI-I, CRI-II, AIP, TyG, and TyG-BMI, all with statistically significant differences (*p* < 0.05). In contrast, HDL-C levels were significantly lower in the MASLD+ASCVD group than in the MASLD-only group (*p* < 0.05).

No statistically significant differences were observed in the remaining variables between the two groups (*p* > 0.05). A summary of baseline characteristics is presented in **Table 1**.

**Table 1:** Baseline characteristics of MASLD patients in the training and validation sets, stratified by ASCVD status.

Characteristics	Training set (n= 413)		*p* ^1^ value	Validation set (n= 177)		*p* ^2^ value	*p* ^3^ value
	MASLD (n = 107)	MASLD+ ASCVD (n = 306)		MASLD (n = 49)	MASLD+ ASCVD (n = 128)		
SEX, n (%)			0.017			0.27	0.067
Female	49 (45.8)	99 (32.4)		17 (34.7)	32 (25.0)		
Male	58 (54.2)	207 (67.6)		32 (65.3)	96 (75.0)		
SMOKE, n (%)			0.101			1	0.32
No	84 (78.5)	213 (69.6)		37 (75.5)	98 (76.6)		
Yes	23 (20.5)	93 (30.4)		12 (24.6)	30 (23.4)		
Fatty liver, n (%)			0.647			0.312	0.985
Grade 1	52 (48.6)	137 (44.8)		22 (44.9)	60 (46.9)		
Grade 2	47 (44.0)	138 (45.1)		20 (40.8)	59 (46.1)		
Grade 3	8 (7.4)	31 (10.1)		7 (14.3)	9 (7.0)		
Age (years)	63 (59, 69)	62 (54, 68)	0.062	63 (55, 68)	64 (57, 69)	0.475	0.356
Fib- 4	1.30 (1.01, 1.62)	1.29 (0.95, 1.81)	0.924	1.29 (0.93, 1.69)	1.39 (1.06, 2.02)	0.056	0.12
BMI (kg/m^2^)	26.63 (24.79, 28.73)	26.99 (25.01, 29.07)	0.362	27.18 (24.91, 29.32)	26.27 (24.2, 28.74)	0.371	0.451
FBG (mg/dL)	102.42 (91.0, 117.72)	110.34 (95.76, 133.33)	0.003	106.56 (98.2, 119.7)	113.94 (95.3, 146.74)	0.34	0.211
AST (U/L)	20 (17, 25)	21 (17, 30)	0.055	21 (17, 26)	22 (18, 34)	0.096	0.313
ALT (U/L)	20 (15, 27)	24 (17, 34)	0.002	21 (18, 30)	25 (17, 37)	0.235	0.175
TG (mg/dL)	116.82 (90.71, 177.44)	145.14 (104.43, 198.24)	0.012	130.10 (94.69, 164.61)	136.29 (98.01, 204.44)	0.299	0.774
TC (mg/dL)	162.16 (127.03, 189.38)	174.13 (142.08, 207.14)	0.003	154.44 (131.27, 176.83)	174.90 (137.36, 210.91)	0.004	0.59
Lp (a) (mg/L)	107 (50, 202)	165 (64, 353)	0.002	115 (57, 237)	166.50 (75.75, 346.75)	0.089	0.911
HDL-C (mg/dL)	50.4 ± 9.6	48.1 ± 10.6	0.043	48.4 ± 11.7	48.6 ± 12.7	0.932	0.569
LDL-C (mg/dL)	84.69 (60.52, 105.18)	99.38 (72.41, 123.74)	< 0.001	81.21 (64.58, 102.48)	97.64 (70.77, 127.71)	0.003	0.762
Scr (mg/dL)	0.96 (0.87, 1.11)	1.03 (0.9, 1.18)	0.006	0.98 (0.83, 1.15)	1.07 (0.92, 1.17)	0.205	0.55
Cys-C (mg/dL)	0.09 (0.08, 0.11)	0.09 (0.08, 0.11)	0.203	0.09 (0.08, 0.11)	0.1 (0.08, 0.11)	0.09	0.451
PLT (10^9^/L)	215 (187, 254)	223 (189, 267)	0.332	222 (177, 259)	223 (197, 256)	0.878	0.289
UA (umol/L)	330 (276.5, 390)	372.5 (306, 441.75)	< 0.001	349 (303, 408)	370.5 (310.25, 446.25)	0.178	0.261
SI	10.4 ± 2.6	10.8 ± 2.6	0.286	10.9 ± 2.9	10.6 ± 2.7	0.56	0.753
CHG	5.08 (4.88, 5.38)	5.31 (5.07, 5.61)	< 0.001	5.27 (4.91, 5.39)	5.39 (5.11, 5.72)	0.004	0.152
CRI-I	3.10 (2.74, 3.71)	3.72 (3.03, 4.37)	< 0.001	3.34 (2.82, 3.92)	3.74 (3.00, 4.46)	0.002	0.533
CRI-II	1.67 (1.29, 2.04)	2.12 (1.58, 2.67)	< 0.001	1.82 (1.34, 2.16)	2.09 (1.55, 2.76)	0.002	0.736
AIP	0.41 (0.23, 0.56)	0.47 (0.31, 0.65)	0.005	0.40 (0.29, 0.62)	0.44 (0.28, 0.67)	0.606	0.955
TyG	8.78 (8.39, 9.20)	9.01 (8.62, 9.40)	< 0.001	8.85 (8.47, 9.27)	9.05 (8.53, 9.48)	0.205	0.619
TyG-BMI	237.25 (216.37, 250.95)	243.72 (221.63, 267.56)	0.024	243.6 ± 35.5	243.2 ± 35.4	0.942	0.981

Data are presented as median (interquartile range, IQR), mean ± standard deviation (SD), or number (percentage), as appropriate. *P* ¹: *P* -value for comparison between MASLD and MASLD+ASCVD groups in the training set. *P* ²: *P* -value for comparison between MASLD and MASLD+ASCVD groups in the validation set. *P* ³: *P* -value for comparison between the training and validation sets (inter-group difference). Abbreviations: Fib-4, Fibrosis-4 index; BMI, body mass index; FBG, fasting blood glucose; AST, aspartate aminotransferase; ALT, alanine aminotransferase; TG, triglycerides; TC, total cholesterol; HDL-C, high-density lipoprotein cholesterol; LDL-C, low-density lipoprotein cholesterol; Scr, serum creatinine; Cys-C, cystatin C; UA, uric acid; Plt, platelet count; SI, sarcopenia index; CHG, cholesterol–HDL–glucose index; CRI-I, Castelli’s Risk Index I; CRI-II, Castelli’s Risk Index II; AIP, atherogenic index of plasma; TyG, triglyceride–glucose index; TyG-BMI, BMI-adjusted TyG index.

### 3.2 Feature selection results

**Figure 2:**
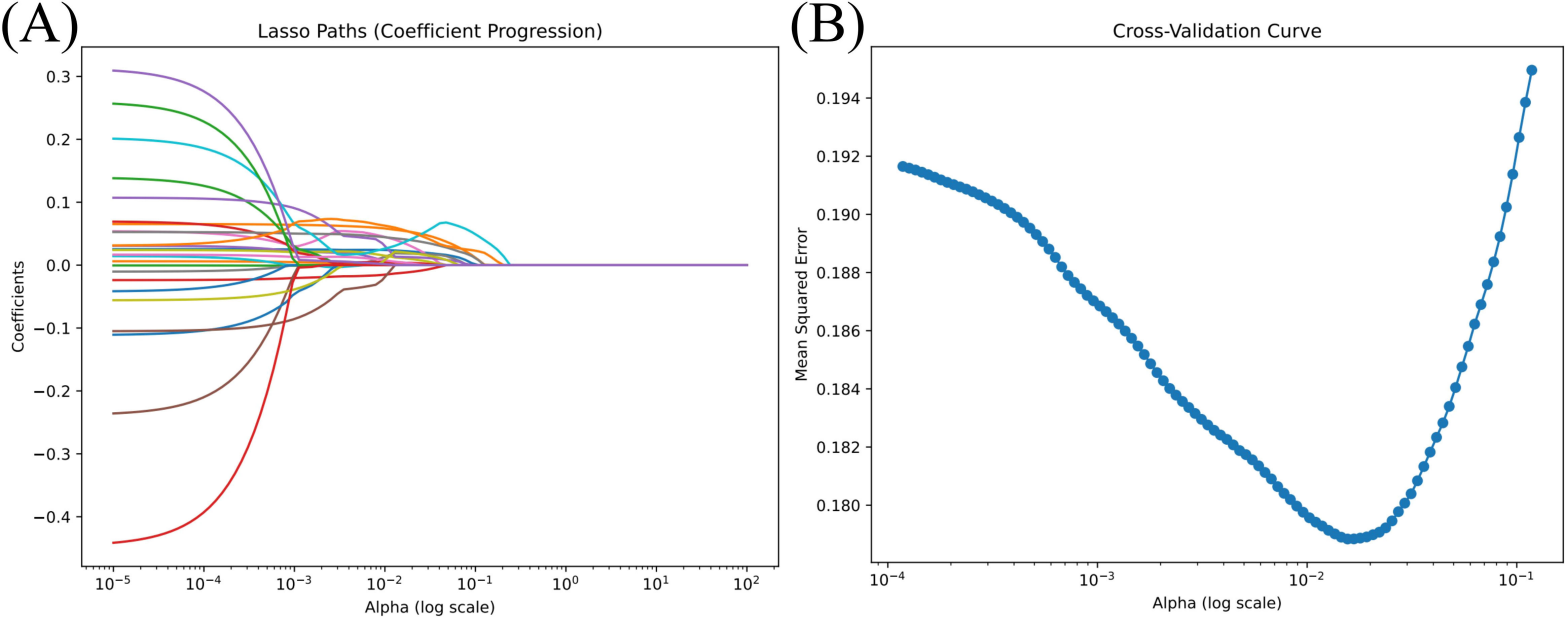
Feature selection process using LASSO regression. **(A)** Coefficient shrinkage paths for all 25 candidate variables using least absolute shrinkage and selection operator (LASSO) regression. As the regularization parameter α (log scale) increases, less informative features are penalized and their coefficients shrink toward zero. **(B)** Ten-fold cross-validation curve showing the relationship between mean squared error (MSE) and α values. The optimal α, corresponding to the minimum MSE, was used to select the final subset of predictive features. This approach balances model simplicity and generalization performance. A total of 25 clinical and biochemical variables were entered into the model. Feature selection and visualization were performed using the LassoCV module from Scikit-learn (Python 3.8).

A total of 25 clinical and biochemical variables were initially collected from the training dataset. To improve modeling efficiency, reduce redundancy, and minimize potential multicollinearity, we applied LASSO regression combined with 10-fold cross-validation for feature selection. As a result, eight variables with strong predictive value were retained (**Figure 2**): CHG, CRI-II, Lp(a), Scr, UA, AST, SI, and sex. These selected features were used in the subsequent modeling process, balancing predictive performance and model simplicity. The regularization path (**Figure 2**) illustrates the progressive shrinkage of variable coefficients as the regularization parameter (λ) increases.

### 3.3 Predictive performance of machine learning models

**Figure 3:**
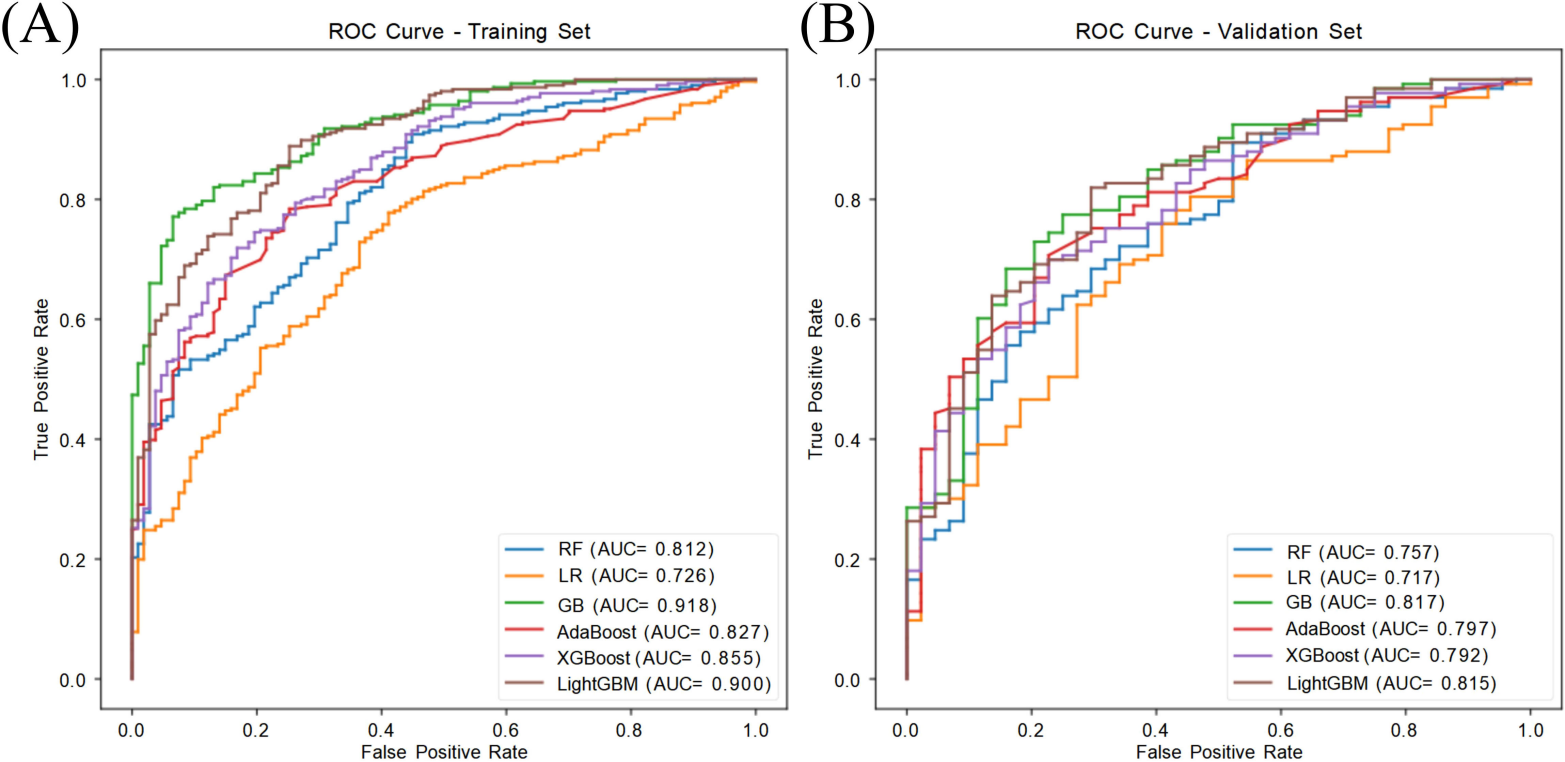
ROC curves of six machine learning models in predicting ASCVD among MASLD patients. **(A)** Training set (n = 413); **(B)** Validation set (n = 177). Among all models, Gradient Boosting (GB) achieved the highest AUC in both sets (AUC = 0.918 in training, 0.817 in validation), indicating superior discriminative performance. Model performance was evaluated using 10-fold cross-validation and plotted with the ROC curve and AUC (95% CI). Abbreviations: RF, Random Forest; LR, Logistic Regression; GB, Gradient Boosting; AdaBoost, Adaptive Boosting; XGBoost, Extreme Gradient Boosting; LightGBM, Light Gradient Boosting Machine.

**Figure 4:**
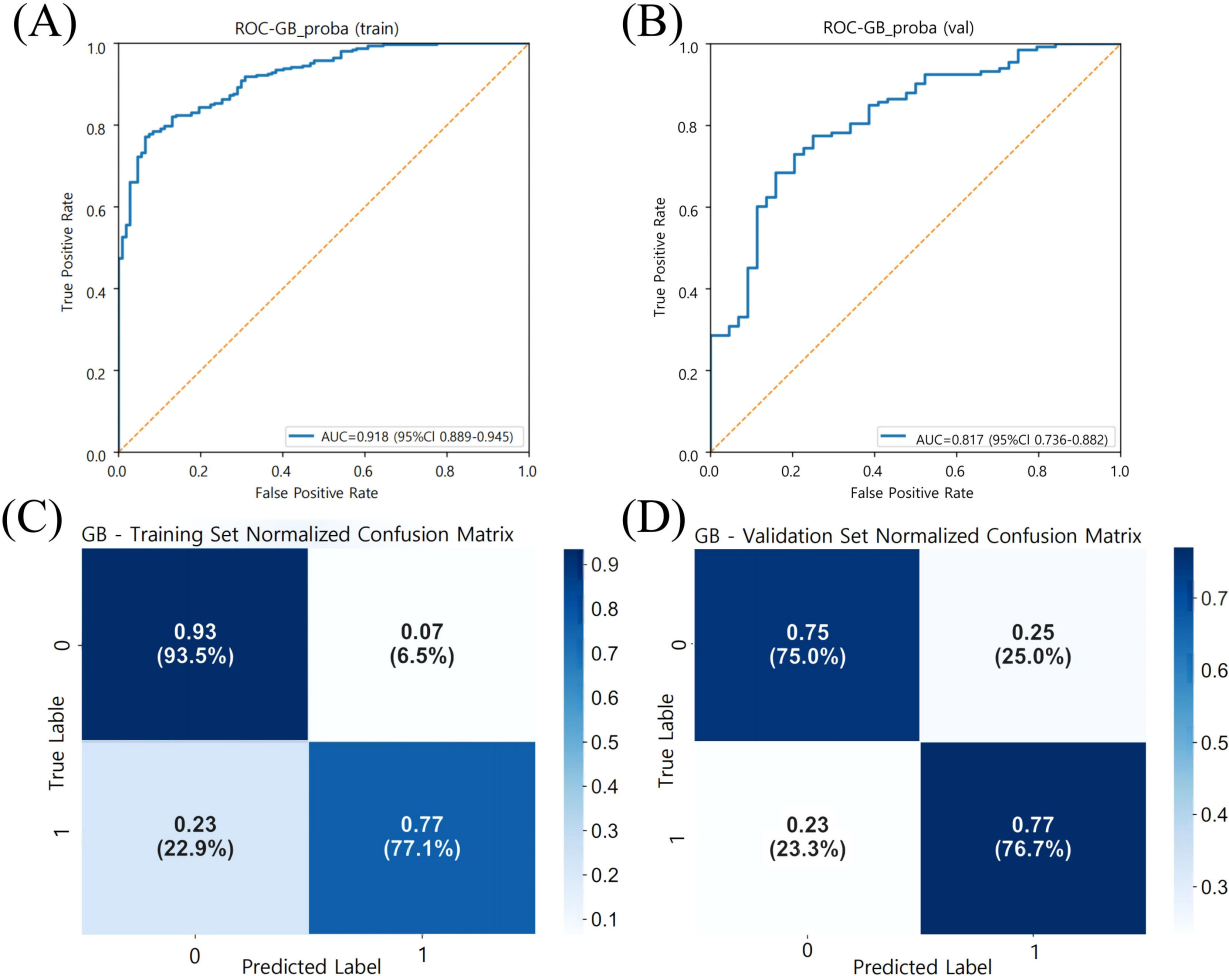
Model Performance of gradient boosting (GB) in training and validation sets. **(A)** ROC curve of the GB model in the training set (n = 413), with an AUC of 0.918 (95% CI: 0.890–0.945). **(B)** ROC curve of the GB model in the validation set (n = 177), with an AUC of 0.817 (95% CI: 0.739–0.883). The orange dashed line represents the no-discrimination reference line (AUC = 0.5). **(C, D)** Normalized confusion matrices of the GB model for the training and validation sets, respectively. Class 0: MASLD without ASCVD; Class 1: MASLD with ASCVD. Color intensity indicates normalized frequency. Model performance was evaluated using 10-fold cross-validation and reported using AUC, sensitivity, and specificity.

All six machine learning models demonstrated favorable predictive performance in both the training and validation cohorts. Among them, the GB model consistently outperformed others, achieving the highest AUC in both the training set (AUC = 0.918, 95% CI: 0.890–0.945) and validation set (AUC = 0.817, 95% CI: 0.739–0.883), as shown in **Table 2** and **Figure 3**. In addition to AUC, the GB model also exhibited superior sensitivity (0.811), specificity (0.725), accuracy (0.785), and F1 score (**Table 3**, **Figure 4**), indicating its robust and balanced classification ability across multiple performance metrics.

**Table 2:** Predictive performance of six machine learning models in the validation cohort.

	AUC	Sensitivity	Specificity	Precision	F1 score
RF	0.757(0.675-0.839)	0.925(0.886-0.964)	0.364(0.293-0.435)	0.815(0.757-0.872)	0.866(0.816-0.916)
LR	0.717(0.633-0.796)	0.812(0.754-0.870)	0.477(0.404-0.551)	0.824(0.768-0.880)	0.818(0.761-0.875)
GB*	0.817(0.739-0.883)*	0.767(0.705-0.829)	0.750(0.686-0.814)	0.903(0.859-0.946)	0.829(0.774-0.885)
AdaBoost	0.797(0.720-0.865)	0.812(0.754-0.870)	0.568(0.495-0.641)	0.850(0.798-0.903)	0.831(0.776-0.886)
XGBoost	0.792(0.717-0.857)	0.759(0.696-0.822)	0.614(0.542-0.685)	0.856(0.804-0.908)	0.805(0.746-0.863)
LightGBM	0.815(0.743-0.881)	0.887(0.841-0.934)	0.500(0.426-0.574)	0.843(0.789-0.896)	0.864(0.814-0.915)

Values are expressed as mean (95% confidence interval). The Gradient Boosting (GB) model achieved the best overall performance, with the highest AUC and balanced sensitivity and specificity. F1-score is a metric synthesizing precision (proportion of positive predictions that are true) and recall (proportion of actual positives correctly predicted), balancing them to comprehensively reflect classification performance. AUC, area under the receiver operating characteristic curve; F1 score: harmonic mean of precision and recall; RF, random forest; LR, logistic regression; GB, gradient boosting; AdaBoost, adaptive boosting; XGBoost, extreme gradient boosting; LightGBM, light gradient boosting machine. "*" denotes highlighting that GB is the optimal model.

**Table 3:** Performance evaluation of the gradient boosting (GB) model in training and validation sets.

Metric	Training set (n=107/306)	Validation set (n=49/128)
AUC	0.918 (0.890-0.944)	0.817 (0.739-0.883)
Sensitivity	0.771(0.731-0.812)	0.767 (0.705-0.829)
Specificity	0.935(0.911-0.958)	0.750 (0.686-0.814)
Precision	0.971(0.955-0.987)	0.903 (0.859-0.946)
F1 score	0.860(0.826-0.893)	0.829 (0.774-0.885)

Model performance metrics for the Gradient Boosting (GB) algorithm were assessed in both the training set (n = 413; 306 positive, 107 negative) and validation set (n = 177; 128 positive, 49 negative). The GB model achieved an AUC of 0.918 in the training set and 0.817 in the validation set, with consistently high sensitivity, specificity, precision, and F1 score across both cohorts. Values are presented as point estimates with 95% confidence intervals in parentheses. Performance was evaluated based on five metrics: area under the receiver operating characteristic curve (AUC), sensitivity (recall), specificity, precision (positive predictive value), and F1 score (harmonic mean of precision and recall).

**Figure 5:**
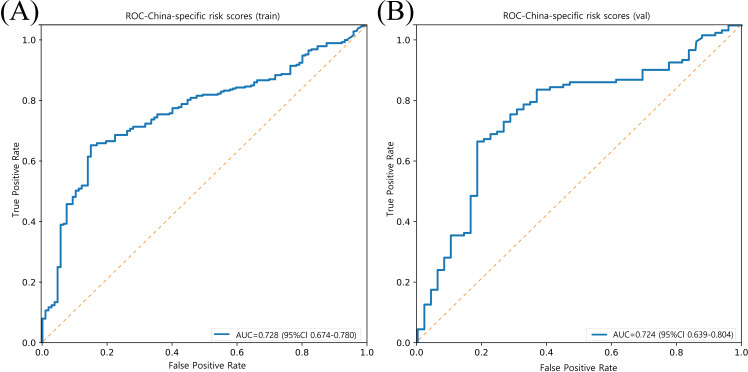
Model performance of prediction for ASCVD risk in China (China-PAR project) in training and validation sets. **(A)** ROC curve of the China-PAR project model in the training set (n = 413), with an AUC of 0.728 (95% CI: 0.674–0.780). **(B)** ROC curve of the China-PAR project model in the validation set (n = 177), with an AUC of 0.724 (95% CI: 0.639–0.804). The orange dashed line represents the no-discrimination reference line (AUC = 0.5).

Compared with Clinical Risk Scores, our model demonstrates superior performance in both the training set (AUC = 0.728, 95% CI: 0.674–0.780) and validation set (AUC = 0.724, 95% CI: 0.639–0.804) (**Figure 5**).

### 3.4 SHAP-based interpretation of the optimal model

To enhance the transparency and clinical interpretability of the machine learning model, SHAP analysis was applied to the best-performing GB model at both the global and individual levels.

**Figure 6:**
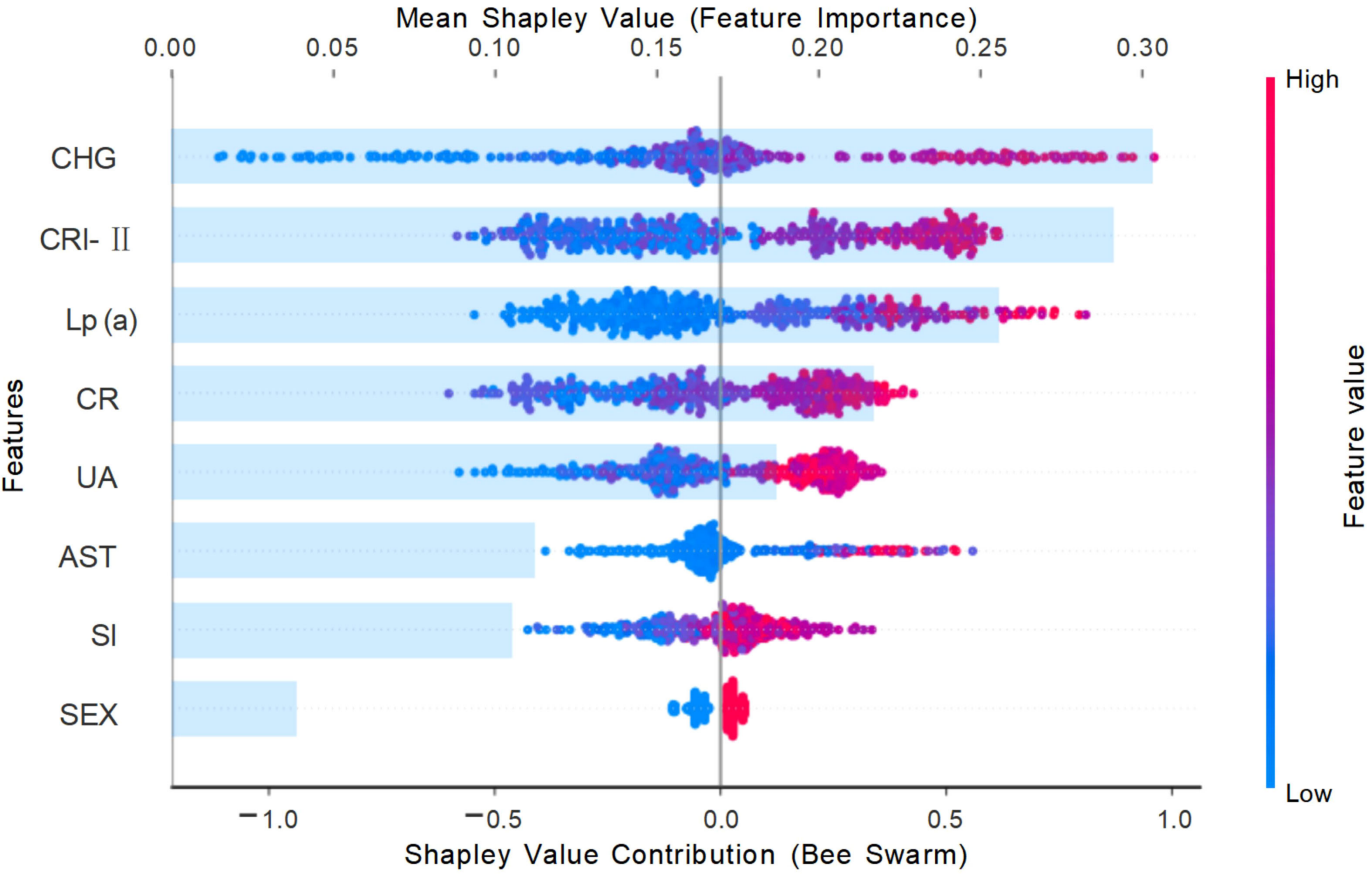
Global feature importance based on SHAP values from the gradient boosting model Each dot represents an individual SHAP value for a feature in one patient. The x-axis indicates the direction and magnitude of the feature’s impact on model output. Features are ranked by their mean absolute SHAP value. The color gradient (blue to red) represents low to high feature values. Features such as CHG, CRI-II, and Lp **(a)** show strong positive contributions to ASCVD risk prediction. SHAP values were derived from the Gradient Boosting model trained on the training set (n = 413), using the SHAP Python package (v0.40.0). Abbreviations: CHG, cholesterol-glucose index; CRI-II, Castelli risk index II; Lp(a) , lipoprotein (a); CR, creatinine; UA, uric acid; AST, aspartate transaminase; SI, systolic index.

**Figure 7:**
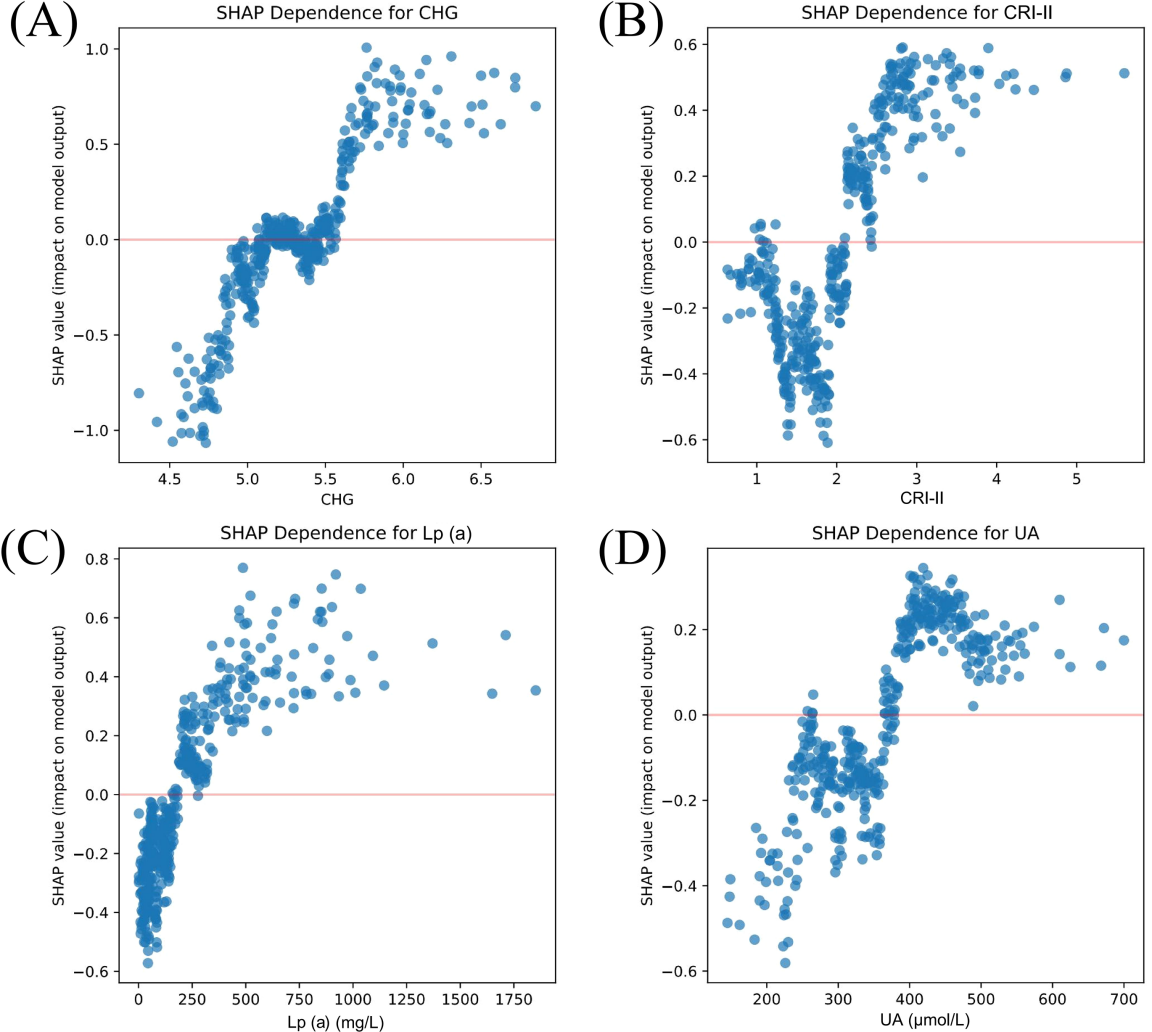
SHAP dependence plots of key predictive features SHapley Additive exPlanations (SHAP) dependence plots showing the marginal effects of the four most influential features on the prediction output of the Gradient Boosting model: **(A)** CHG, **(B)** CRI-II, **(C)** Lp(a), and **(D)** UA. The x-axis represents the actual value of each feature. The y-axis shows the corresponding SHAP value, indicating the direction and magnitude of that feature’s contribution to the model prediction. Each dot corresponds to a single patient. These plots reveal nonlinear, positive associations between the feature values and their impact on ASCVD risk prediction, particularly highlighting threshold effects for CHG and Lp(a). The SHAP values were derived using the Gradient Boosting model trained on 413 patients in the training cohort, using the SHAP Python package (v0.40.0). CHG, cholesterol–glucose index; CRI-II, Castelli risk index II; Lp(a), lipoprotein (a); UA, uric acid.

At the global level, the SHAP feature importance plot (**Figure 6A**) identified CHG, CRI-II, Lp(a), Scr, and UA as the top five most influential predictors of ASCVD. The SHAP summary plot (**Figure 6B**) illustrated the direction and magnitude of each feature’s contribution to the prediction outcome. Specifically, higher values of CHG, CRI-II, Lp(a), and UA were associated with greater positive SHAP values, indicating stronger driving forces toward predicting ASCVD. In addition, SHAP dependence plots (**Figure 7**) demonstrated a consistent positive marginal effect of these features on the model output, suggesting nonlinear trends and potential threshold effects.

**Figure 8:**
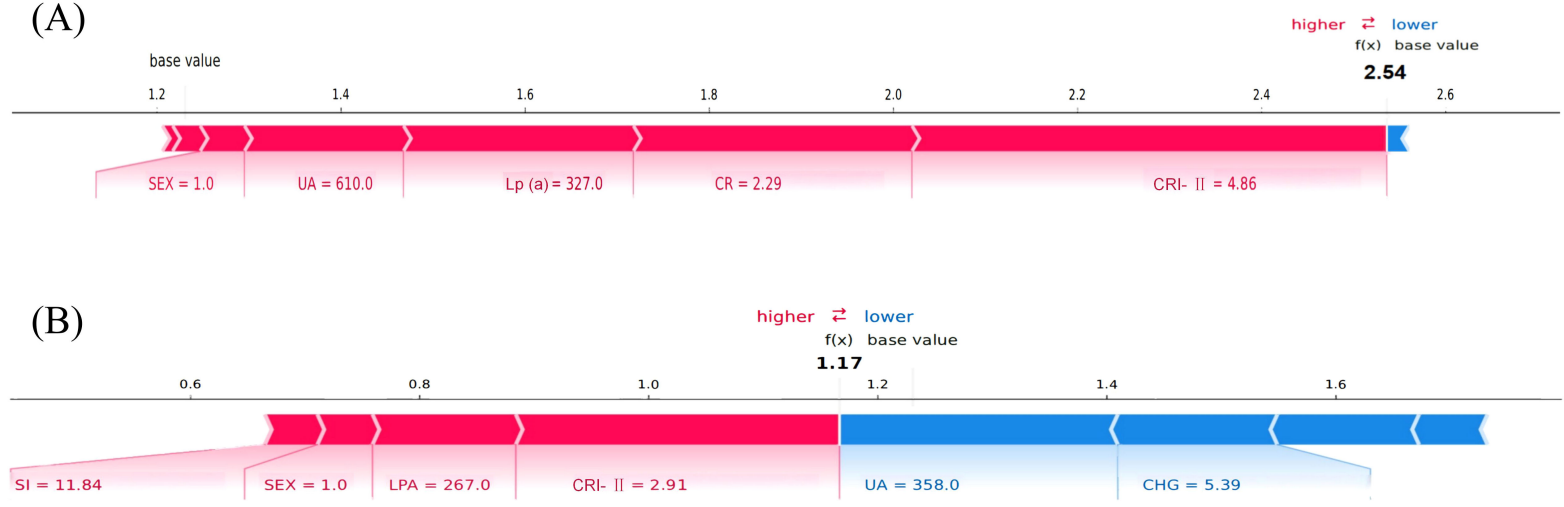
SHAP force plots illustrating individual prediction explanations by the gradient boosting model. **(A)** Force plot for Patient A (true positive). The final model output f(x) = 2.54 exceeds the base value, indicating a high risk of ASCVD. **(B)** Force plot for Patient B (true negative). The output f(x) = 1.17 remains below the base value, suggesting no ASCVD. Red bars represent features that increase the predicted risk (positive contribution), while blue bars represent features that decrease it (negative contribution). The length of each bar indicates the magnitude of contribution. The grey vertical line represents the model’s base value, i.e., the expected output when no features are present. SHAP (SHapley Additive exPlanations) values were calculated using the SHAP Python package (v0.40.0), based on the Gradient Boosting model trained on the training cohort (n = 413).

At the individual level, force plots were generated for two representative patients—one correctly predicted as ASCVD-positive (true positive) and the other as ASCVD-negative (true negative) (**Figures 8A, B**). In these visualizations, red bars denote features that increase the prediction probability for ASCVD, while blue bars indicate features that decrease it. The length of each bar reflects the magnitude of contribution, and the final output is determined by the cumulative effect of all features starting from the model’s base value. For instance, markedly elevated CHG and Lp(a) levels were major contributors to the ASCVD prediction, whereas higher HDL-C levels played a protective role.

In summary, the SHAP analysis not only confirmed the strong predictive value of key metabolic features in the GB model but also provided individualized explanations for model outputs. This highlights the model’s potential utility in clinical decision support, offering both accurate prediction and interpretability.

## 4 Discussion

This study, based on real-world data from hospitalized MASLD patients, developed and compared multiple machine learning models to classify concurrent ASCVD. Eight key predictive features were identified through LASSO regression. The GB algorithm demonstrated superior predictive performance and stability within our cohort of hospitalized MASLD patients​, with consistent results across both the training and validation sets. However, its relative advantage may vary across different clinical populations or with inclusion of additional features, underscoring the need for external validation to ensure broader applicability.​ Furthermore, the SHAP method was employed to enhance the interpretability and clinical transparency of the model.

MASLD and ASCVD share a wide range of metabolic risk factors. Our findings revealed that patients in the MASLD+ASCVD group exhibited significantly more severe abnormalities in metabolic indicators, indicating a substantial overlap between atherosclerotic risk and hepatic metabolic dysregulation. In the SHAP feature importance ranking, CHG, CRI-II, Lp(a), Scr, and UA emerged as the top contributors to the predictive model. These features reflect key metabolic mechanisms such as IR, dyslipidemia, renal dysfunction, and systemic inflammation, which are consistent with the established pathophysiology of both MASLD and ASCVD (8).

Traditional single lipid markers such as LDL-C have demonstrated limited effectiveness in early ASCVD classification of prevalent ASCVD (28). A previous study reported that approximately 77% of hospitalized ASCVD patients had LDL-C levels within the normal range (29), suggesting its insufficient standalone predictive value. In contrast, composite lipid indices such as CRI, AIP, and the TyG index, which simultaneously reflect multiple metabolic pathways, have shown superior performance in predicting cardiovascular and metabolic diseases (13, 30–32). Notably, this study is the first to systematically evaluate the CHG index for identifying ASCVD risk among MASLD patients, expanding its potential application in early metabolic risk management.

Unlike many prior “black-box” models, this study emphasized model interpretability. At the population level, SHAP feature rankings and dependence plots revealed the marginal and non-linear effects of key variables. At the individual level, SHAP force plots provided transparent explanations of the prediction logic for specific patients, highlighting the positive or negative impact of each variable. This approach offers valuable insight for developing transparent clinical decision support systems (CDSS).

Despite the promising findings, several limitations must be acknowledged. First, this study was conducted as a single-center retrospective analysis. The cohort was intentionally enriched with high-risk MASLD patients from tertiary hospital cardiology clinics, resulting in a higher observed ASCVD prevalence (73.6%) than population-based samples.​​ While this design ensured adequate event rates for predictive modeling, it may introduce selection bias and limit external generalizability to community-based primary prevention settings. External validation in broader and more diverse cohorts is therefore warranted. Second, our outcome definition relied on administrative ICD codes (e.g., I50.x for heart failure), which cannot distinguish between atherosclerotic and non-atherosclerotic etiologies of congestive heart failure. Prospective studies with protocol-defined ASCVD adjudication are needed to confirm etiology-specific classifications. Third, although L1 regularization was used to mitigate overfitting, the relatively small sample size still poses a risk to the robustness of the conclusions. Fourth, the current model does not incorporate multimodal data such as imaging scores, genomic information, or lifestyle factors, which could further enhance predictive accuracy. Lastly, while SHAP-based explanations improve interpretability, further validation through mechanistic studies and clinical pathways is needed to confirm their clinical applicability and acceptance.

In conclusion, this study successfully developed a high-performing and interpretable classification model for identifying ASCVD in MASLD patients. By integrating machine learning with SHAP-based explanations, the model demonstrates strong potential for precision stratification and individualized risk assessment in metabolic diseases. The model demonstrates strong predictive performance in treatment-naive MASLD patients, providing a novel tool for early high-risk identification.​ While excluding lipid-lowering or antiplatelet therapy may limit immediate generalizability to medicated populations, this approach helps preserves the integrity of metabolic risk drivers analysis. Importantly the key biomarkers highlighted by SHAP analysis—such as (e.g., Lp(a)—remain clinically actionable therapeutic targets for clinical intervention.​​ The superior performance of our model compared to the China-PAR score underscores the value of a precision medicine approach for the distinct high-risk MASLD population. This difference likely stems from a fundamental divergence in design intent: while established scores like China-PAR are optimized for general population screening, our model is specifically engineered to capture the unique risk profile of MASLD patients. Therefore, our work provides a complementary, population-specific tool rather than a direct replacement for general-purpose scores, enabling more refined risk stratification for this cohort. Future work should focus on expanding to multicenter, multimodal datasets, conducting performance comparisons with traditional risk assessment models, developing integrated risk engines combining traditional factors with MASLD-specific biomarkers, and exploring the feasibility of clinical deployment to support personalized cardiovascular risk management in MASLD.

## 5 Conclusion

This study developed and validated multiple machine learning models to classify concurrent atherosclerotic cardiovascular disease (ASCVD) in hospitalized MASLD patients, using real-world cross-sectional data.​ Among the evaluated methods, tree-based ensemble learning algorithms—particularly Gradient Boosting​​—demonstrated the most robust performance in this specific cohort, showing high predictive accuracy and stability across both the training and validation sets. The eight high-value features selected via LASSO regression were also confirmed to have strong predictive contributions through SHAP interpretability analysis.

The innovation of this study lies in the integration of high-performance prediction modeling with interpretable machine learning, balancing accuracy with clinical applicability. In particular, the application of SHAP provided transparent decision-making support at both global and individual levels, enhancing the model’s practical value in CDSS.

This model provides a novel tool for identifying MASLD patients with prevalent ASCVD, enabling individualized clinical assessment during hospitalization. ​Future work should focus on optimizing the model using multicenter datasets and expanding its scope by incorporating imaging, genetic, and behavioral data. This would provide a more comprehensive tool for early identification and intervention in MASLD patients at high risk for ASCVD.

## Data Availability

The original contributions presented in the study are included in the article/**Supplementary Material**. Further inquiries can be directed to the corresponding author.
