# Supplementary material for: Explainable machine learning model for classifying atherosclerotic cardiovascular disease in patients with metabolic dysfunction-associated steatotic liver disease
Source: Front Endocrinol (Lausanne). 2025 Oct 30;16:1684558. doi: 10.3389/fendo.2025.1684558 (PMC12611696; doi:10.3389/fendo.2025.1684558)
Supplement: Supplementary file 1 [file SupplementaryFile1.docx]

**Appendix A: Reproducibility Specifications**

**A.1 Missing Values**

The dataset contained no missing values across all analyzed features. This was confirmed through:

Comprehensive null-check: pd.isnull().sum().sum() == 0

Data source validation: EHR extraction criteria required complete fields for all clinical variables

**A.2 Random Seeds**

Data splitting: seed=42(set during train-test split)

Hyperparameter search: random_state=42(set in RandomizedSearchCV)

Model initialization: Library default random states were used

**A.3 Hyperparameter Configurations**

# ================ Random Forest (rb) ================

{

'rb__max_depth': [2, 3],

'rb__n_estimators': [50, 100],

'rb__min_samples_split': [10, 15],

'rb__min_samples_leaf': [2, 4],

'rb__max_features': ['sqrt', 'log2']

}

# ================ Logistic Regression (lr) ================

{

# Default parameters used (no hyperparameter tuning)

}

# ================ Gradient Boosting (gbc) ================

{

'gbc__n_estimators': [50, 100],

'gbc__learning_rate': [0.01, 0.05],

'gbc__max_depth': [2, 3],

'gbc__min_samples_split': [15, 20],

'gbc__min_samples_leaf': [6, 8],

'gbc__subsample': [0.6, 0.7]

}

# ================ AdaBoost (abc) ================

{

'abc__n_estimators': [50, 100],

'abc__learning_rate': [0.01, 0.05]

}

# ================ XGBoost (xgb) ================

{

'xgb__n_estimators': [50, 100, 200, 300],

'xgb__max_depth': [2, 3, 5],

'xgb__learning_rate': [0.01, 0.05],

'xgb__subsample': [0.7, 0.8],

'xgb__colsample_bytree': [0.7, 0.8],

'xgb__reg_alpha': [0, 0.1],

'xgb__reg_lambda': [0, 0.1]

}

# ================ LightGBM (lgb) ================

{

'lgb__n_estimators': [50, 100, 200, 300],

'lgb__max_depth': [2, 3, 5],

'lgb__learning_rate': [0.01, 0.05],

'lgb__num_leaves': [10, 20],

'lgb__subsample': [0.7, 0.8],

'lgb__colsample_bytree': [0.6, 0.7],

'lgb__reg_alpha': [0, 0.1],

'lgb__reg_lambda': [0, 0.1]

}

**Search Configuration:**

Algorithm: RandomizedSearchCV

Iterations: 50per model

Cross-validation: 5-fold

Scoring metric: roc_auc

Parallelization: n_jobs=-1(all available cores)

**A.4 Software Environment**

| **Library** | **Version** |
| --- | --- |
| Python | 3.8 |
| scikit-learn | 0.24.2 |
| NumPy | 1.21.2 |
| pandas | 1.3.3 |
| Matplotlib | 3.4.3 |
| XGBoost | 1.5.1 |
| LightGBM | 3.3.2 |
| SciPy | 1.7.1 |
| joblib | 1.1.0 |

This appendix contains full reproducibility specifications requested by reviewers, including hyperparameter search spaces, random seed configurations, and software environment details.
